# Impact of Nurses’ Intervention in the Prevention of Falls in Hospitalized Patients

**DOI:** 10.3390/ijerph17176048

**Published:** 2020-08-20

**Authors:** Raimunda Montejano-Lozoya, Isabel Miguel-Montoya, Vicente Gea-Caballero, María Isabel Mármol-López, Antonio Ruíz-Hontangas, Rafael Ortí-Lucas

**Affiliations:** 1 Escuela Enfermería La Fe, Valencia (Spain), adscript center of Universitat de Valencia, Research Group GREIACC, Health Research Institute La Fe, 46026 Valencia, Spain; montejano_rai@gva.es (R.M.-L.); demiguel_isa@gva.es (I.M.-M.); marmol_isa@gva.es (M.I.M.-L.); ruiz_anthon@gva.es (A.R.-H.); 2 Public Health Department, Catholic University of Valencia, 46001 Valencia, Spain; ortilucas@gmail.com

**Keywords:** accidental falls, hospitalization, patient safety, accident prevention, nursing education research

## Abstract

Background: Clinical safety is a crucial component of healthcare quality, focused on identifying and avoiding the risks to which patients are exposed. Among the adverse events that occur in a hospital environment, falls have a large impact (1.9–10% of annual income in acute care hospitals); they can cause pain, damage, costs, and mistrust in the health system. Our objective was to assess the effect of an educational intervention aimed at hospital nurses (systematic assessment of the risk of falls) in reducing the incidence of falls. Methods: this was a quasi-experimental study based on a sample of 581 patients in a third level hospital (Comunitat Valenciana, Spain). An educational program was given to the intervention group (*n* = 303), and a control group was included for comparison (*n* = 278). In the intervention group, the nurses participated in a training activity on the systematized assessment of the risk of falls. Analysis was undertaken using the Bayesian logistic regression model. Results: a total of 581 patients were studied (50.6% male, 49.4% female), with an average age of 68.3 (DT = 9) years. The overall incidence of falls was 1.2% (0.3% in the intervention group and 2.2% in the control group). Most of the falls occurred in people ≥65 years old (85.7%). The intervention group had a lower probability of falling than the control group (OR: 0.127; IC95%: 0.013–0.821). Neither the length of hospital stay, nor the age of the participants, had any relevant effect. Conclusions: the systematic assessment of the risk of a patient falling during hospital processes is an effective intervention to reduce the incidence of falls.

## 1. Background

Clinical safety is a crucial component of healthcare quality that focuses on identifying and avoiding the risks to which patients are exposed in their relationship with the healthcare system, and whose materialization is known as Adverse Events (AEs) in the international literature. AEs cause significant morbidity and mortality, and consequently trying to avoid them, or at least to reduce them, is a priority for health institutions. Background information shows that almost half of falls are avoidable. To achieve this, systems must be designed to make it easier to carry out processes properly [1,2].

Among the AEs that occur in a hospital environment, falls cause a significant impact, because it is a type of accident that reflects system failures in organizational structures and processes. Studies have shown that falls cost between 1.6% and 13.4% of the annual income in acute care hospitals [3,4,5,6,7]. Reported rates range from 1.3 to 8.9 falls/1000 inpatient days in acute care hospitals (30% of these resulting in serious injury) [8,9,10]. Although they might not always provoke serious harm, there are instances that require intervention as a result of the pain and suffering caused to the patient and their relatives [3,6]. Costs resulting from falls alone have been reported at between 0.85% and 1.5% of the total health care expenses within the United States, Australia, the European Union, and the United Kingdom [5]. According to the World Health Organization (WHO), these financial costs are additional to the costs of damage to people and mistrust in the health system; therefore, health institutions should seek to eradicate avoidable falls [4,11].

The literature shows that those who fall are people with limited mobility, an altered state of consciousness, advanced age, and sensory deficits. When several risk factors are present at the same time, the risk is much higher [7,12,13,14,15].

Many studies about falls agree that assessing their frequency and identifying risk factors helps to prevent and/or reduce them [15,16,17,18]; thus, in a systematic review that included four meta-analyses on 19 studies on falls, it was shown that programs and interventions focused on hospitalized patients reduce the relative risk of falls by up to 30% [19].

Several research studies have shown that the use of scales to identify patients with a high risk of falls is effective, as it achieves a reduction in falls and a decrease in the injuries that result [3,13,15,16,20,21,22]. In the study by Kobayashi et al. [3], the risk of falls was evaluated using a Fall Assessment Score Sheet at admission and during hospitalization. The authors showed that the incidence of serious events and falls was significantly higher in patients with a higher risk of falling (*p* < 0.05). Bittencourt et al. [13] carried out a transversal study in clinical and surgical stay units using sociodemographic and clinical forms, as well as the MORSE scale, for data collection. They found a significant association (*p* < 0.001) between high risk of falling and neurological clinical hospital stay, trauma surgery, and comorbidities such as diabetes mellitus, systemic arterial hypertension, visual difficulty, dizziness, and fear of falling. Severo et al. [15] developed and validated the SAK fall risk scale; the scale includes seven variables: disorientation/confusion, frequent urination, walking limitations, lack of caregiver, postoperative status, previous falls, and number of medications administered within 72 h prior to the fall. Finally, Hernandez-Herrera et al. [16] designed a checklist of 60 intervention activities for “Fall Prevention” (based on the Nursing Intervention Classification, NIC). The most frequently performed activities were those related to a risk factor, transfer, and patient education.

The study of Pasa et al. [21] concludes that the use of the MORSE assessment scale to identify patients at high risk of falling is effective. They also found an association between a greater number of falls and patients staying longer in hospitals. The work of Hou et al. [22] shows the advantages of applying a tool to identify patients at high risk of falling, which allows nurses to have more control over them.

Nurses, as the professionals responsible for performing assessments upon admission to the hospital, are in an optimal position to identify patients at risk and implement fall prevention programs. Involving this group of professionals (including their leaders) in a culture of responsibility, and improving their training in preventive programs, allows very positive results to be obtained [8,9,23,24].

### 1.1. Framework

Our theoretical framework was based on the systematized assessment of Virginia Henderson’s model of care. This model is considered an axiom for nursing care. The author defines the individual as a whole with fourteen basic needs; among them the need to protect the safety of the person (“to avoid danger”, according to the author). This need, and more specifically the prevention of falls, is the focus of our intervention. The fact of reducing or preventing falls is properly framed within the improvement of patient safety. The role of the nurse, therefore, consists of helping the person to recover their independence when they lack strength, knowledge or will, within this framework of improving safety [25,26,27].

### 1.2. Justification

Based on the dimension of the problem, as well as the consequences of falls (pain, injuries, complications, costs, and increase in hospital stay), we consider it necessary to implement studies with interventions, in order to increase the evidence available around what practices a nurse should implement to reduce the problem.

We proposed as a study hypothesis: patients admitted to units whose nurses have been trained in the systematic assessment of the risk of falls will fall less than those in units in which nurses have not received specific training.

The question is whether the implementation of an advanced and systematized assessment by the nurse following the patient’s admission to a hospital unit reduces the incidence of falls, compared to a traditional assessment.

### 1.3. Objectives

The general objective was to assess the effect of an educational intervention aimed at hospital nurses (systematic assessment of the risk of falls) in reducing the incidence of falls.

## 2. Methods

### 2.1. Study Design

This was a quasi-experimental study with a non-randomized control group.

### 2.2. Population

The study was carried out in 2015 in a third level hospital in the Comunitat Valenciana (Spain). Four Care Units with the highest average patient stay were chosen in Neurology/Neurosurgery, General Internal Medicine, Nephrology/Vascular Surgery, and Traumatology/Urology. Two groups were formed (intervention and control), made up of two Care Units each, so that in both there were patients from both medical and surgical specialties. The assignment of each group was random.

Finally, the intervention was performed in one of the groups (intervention group, formed by the units of Nephrology/Vascular Surgery and Traumatology; the control group was formed by Internal Medicine and Neurology/Neurosurgery). In this sense, the patients were not randomized, but taken from the hospital units where the nurses were trained (or not).

In each group, it was stipulated that a necessary minimum sample size of 258 patients was required for a confidence level of 95% and a statistical power of 80%, estimating an approximate incidence of AEs of 16% in the control group and 8% in the intervention group.

Inclusion Criteria: patients who were admitted during the study period (Neurology/Neurosurgery, General Internal Medicine, Nephrology/Vascular Surgery, and Traumatology/Urology units), with a minimum stay of five days in the unit (this time allowance was estimated to be sufficient to produce the AE object of study). The sample selection was carried out prospectively and consecutively after the training activity, covering patients who met the inclusion criteria to reach the assigned number.

Exclusion Criteria: patients who had dementia or delirium were excluded.

A total of 593 patients were studied, of which 12 patients were excluded (four due to recording errors, and eight who refused to participate in the study). No deaths or dropouts occurred during the study.

### 2.3. Study Assessment Parameters

Independent variables:

- Sex (men, women);
- Age categorized (in years) and age groups (15–50, 51–64, 65–79, ≥80);
- Nursing units (General Internal Medicine, Neurology and Neurosurgery, Traumatology and Urology, Vascular Surgery and Nephrology);
- Group (control and intervention);
- Type of nurse assessment on admission (traditional method, systematized method);
- Assessment of the risk of falls on admission according to the Downton scale (yes/no) [28] (this scale assesses factors related to the risk of falling, such as sensory deficits, mental state impairment, wandering, and intake of medication whose side effects may influence the occurrence of falls);
- Length of hospital stay in days and in two categories (0–7 and ≥8 days);
- Degree of mobility (non or impaired, unaided in and outside of the room and bathroom);
- Surgical intervention (yes/no);
- Altered consciousness (yes/no);
- Nutritional status on admission according to the Mini Nutritional Assessment-Short Form (MNA-SF) (risk and/or malnutrition and good nutritional status) [29];
- Supply of oxygen (yes/no);
- Has catheters (vascular access; nasogastric tubes; urinary catheterization) and categorized (does not have catheters, has a catheter, and has two or more catheters).

### 2.4. Procedure

Before the start of the study, the necessary tools were developed for each phase of the project: the protocol containing the data collection procedure for the evaluation team, the form with data content to be filled in, and the systematic nurse assessment registry to deploy in the intervention units.

The study was carried out over 8 months, following three phases.

In the first phase (before the quasi-experimental study; this phase lasted two months), a pilot test was carried out using a baseline test which allowed a diagnosis to be made and determined how the information was to be collected to consolidated.

In the second phase (one month), the nurses were trained through programmed theoretical and practical sessions, as well as training reinforcement sessions.

The intervention originally consisted of a formative activity directed to the nurses of the intervention the group. A total of 33 professionals attended (84.6% of the total of nurses of the intervention group). The training workshop was held with two theory and practice sessions of 4 h each, offering the possibility to repeat the workshop upon the nurse´s request to reinforce aspects as necessary. Before starting the formative activity, the attendees were requested to undertake a self-assessment to estimate their level of knowledge about the relevant topics. At the end of the training, the same self-assessment was repeated, resulting in a very positive comparison.

The formative activity focused mainly on the first stage of the nursing process: the assessment [24] was framed in the Human Needs Model of Virginia Henderson, as it is considered the most appropriate to the idiosyncrasies of the institution [24,25,26]. The systematized evaluation was a regulated process that collected all patient information in a bio-psycho-social way (holistic image of the person). This was undertaken at the patient’s admission and continued throughout the care process, which made it possible to identify potential problems and risks and implement their care plan. In the control group, a traditional assessment was undertaken that did not follow a standardized method, and it was intuitive, improvised, and not systematically reflected in the patient’s clinical history.

Finally, the third phase was data collection (five months). This period was needed to reach the pre-set sample.

After a systematic, exhaustive, and complete evaluation of the patients, carried out in the Hospital units of the intervention group, the care plan was optimized (as a result of detecting risks that would not have been detected with the usual evaluation). Afterwards, a follow-up process was initiated in all the units. The established criteria were to conduct a review of the clinical history of the patient, followed by inspection and an interview with him/her and/or family and professionals responsible for his/her care, asking about the incidence of falls.

Blinding was kept simple by not informing patients of the type of assessment received.

### 2.5. Statistical Analysis

Data were registered into a database and analyzed with the statistical program Statistical Package for Social Science (SPSS) version 20.0 (IBM Corporation, Armonk, NY, USA) and R version 3.5.1. (R Core Team, Vienna, Austria).

A descriptive study was performed by assessing the variables related to the total sample and the established groups (control and intervention). The incidence of falls was assessed, concerning the studied variables. The categorical variables are presented in frequencies and percentages, and continuous variables in averages with standard deviations (SD).

Afterward, to assess the probability of falls between the two studied groups, a Bayesian logistic regression model was used. An attempt was made to reduce overfitting by selecting the fewest number of possible variables; the model was adjusted by entering, as confounding factors, the stay in days and the age in years, calculated by the Odds Ratio (OR) with a Credible Interval (CI) of 95%.

### 2.6. Ethical Considerations

The study protocol was approved by the Research Ethics Committee of the Hospital, before implementation of the study. All persons involved were informed and asked for voluntary participation. The data obtained were handled following the law prevailing at that time: the Data Protection Law 15/1999, and the Law 41/2002. Personal data were guarded carefully by the investigation team. The researchers declare they have no ethical conflicts, nor have received any grant or economic benefit for this study.

## 3. Results

### 3.1. Description of the Sample

The sample was a total of 581 patients (response rate = 97.97%), 50.6% men and 49.4% women, with an average age of 68.3 ± 9 years. The control group was 278 patients distributed between the General Internal Medicine unit (23.9%) and Neurology/Neurosurgery (23.9%). The intervention group with a total of 303 patients from the Traumatology and Urology units (35.5%), as well as Vascular Surgery and Nephrology (16.7%).

The average length of stay was 12.2 ± 9 days, and this was lower in the intervention group (10.9 ± 7.5 days) than in the control group (13.7 ± 0.2 days). Two-thirds (66.3%) of the patients belonging to the intervention group were assessed in a systematic way, applying the Downton scale (assessment of falls risk), compared to 2.9% in the control group (Table 1).

### 3.2. Incidence of Falls

Only 1.2% of the patients suffered any falls during the study period. Seven falls were reported: 1 in the intervention group and 6 in the control group, resulting in an incidence of 0.3% and 2.2%, respectively. A higher number of falls were observed in men (85.7%), in persons older than 65 years (85.7%), and in those who stayed more than 7 days in hospital (85.7%). The total number of people who suffered a fall was autonomous in terms of mobility and having some type of catheter (Table 2).

### 3.3. Regression Model

By using the logistic regression model, it was possible to demonstrate that patients in the intervention group had a lower likelihood of falls than those in the control group (OR: 0.127; IC95%: 0.013–0.821). This hypothesis was reinforced with a probability of 0.99, associated with an evidence ratio of 77.43. On the other hand, neither the length of hospital stay, nor the age of the participants in the study had any relevant effect (Table 3).

**Figure 1:**
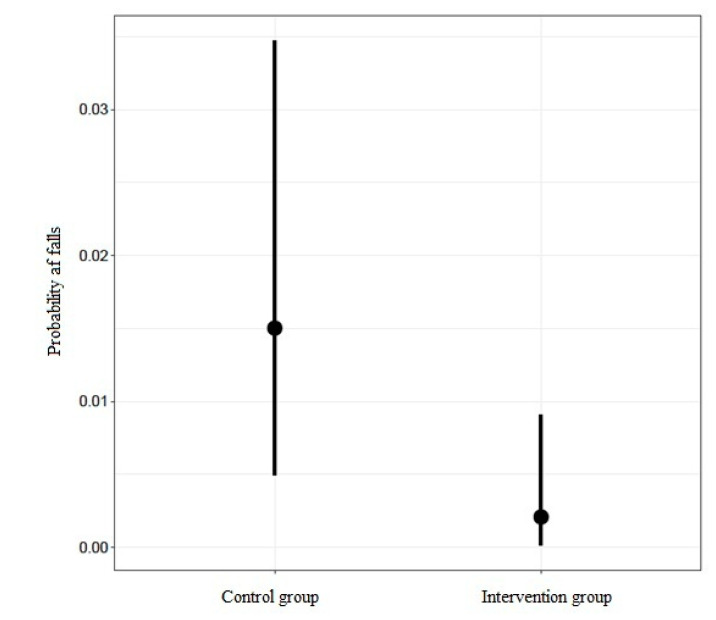
Differences in the probability of fall between the two groups, intervention and control.

Figure 1 shows the partial effect of the group regarding the probability of falls. The dots represent the estimated average probability for each group, while the vertical lines indicate the interval corresponding to that estimate. As can be seen, the probability of a fall in the intervention group was lower than in the control group.

No significant association was found between the intrinsic risk factors and the incidence of falls.

## 4. Discussion

The total incidence of falls was 1.2%. This result was better than in other studies we consulted, with falls affecting between 1.6% and 13.4% of the annual income in hospitals for acute patients [3,4,5,6,7,30,31].

The characteristics of people who suffered a fall correspond in many ways with those described in the reviewed literature. There is evidence that relates advanced age to falls [7,14,32,33,34]. In our study, 85.8% of people that fell were 65 years and older, and 85.7% of those who fell were men; we found similar percentages in a few studies [7,18].

We want to emphasize that all people who suffered falls in our study had a catheter; this is a factor we did not find described by other authors. Regarding the average stay of the patient in the hospital and its influence on the risk of suffering falls [12,13,22,35], 85.7% of falls occurred in patients with stays longer than one week. Luzia et al. [7] reported that 63.2% of patients fall between the 10th and 24th days of hospitalization.

Patients with some level of mobility suffer the most falls according to various authors [12,15,18,22,32]; in our case all people who suffered a fall were autonomous or had a certain level of mobility. This is in agreement with the work of Lopez-Soto et al. [36], which demonstrated that more falls occur while patients are standing or sitting, when entering/leaving the room, and when getting up or getting out of bed. A systematic review of Laguna et al. [37] concludes that the leading causes of falls are related, in addition to age, to preoperative and postoperative status, neurological diseases, and medication. It is known that surgical patients have a higher risk of falling [7,37,38]. In the studied sample, the number of patients with the risk factor of surgical intervention was five times higher in the intervention group; despite this, there was only one fall in surgical patients in the intervention group, which reinforces the benefit of the educational program.

An altered state of consciousness, although it is an intrinsic risk factor identified frequently in the consulted bibliography [7,13,16,30,32,39], was not associated with any falls in patients with this type of problem in our study.

The difference in falls incidence among the studied groups (0.3% in the intervention versus 2.3% in the control group) led us to consider the beneficial effect produced by the systematic method of assessment used by the majority of nurses in the intervention group who, in addition, applied Downton scale [28] to detect the risk of patient falls on admission. The analysis of the intervention by logistic regression revealed that a lower likelihood of falls in the intervention group was associated effectively with the method of care in the units included in this group, with no other studied variables being relevant. Our results are consistent with other studies that advocate for the benefits of using scales to identify the risk of falls [3,13,15,16,20,21,22]. Likewise, the systematic review by Miake-Lye et al. [19] expresses the benefits of programs that include interventions to identify risk factors associated with falls in acute care environments. A study on the incidence of falls in hospitals and nursing homes asserted that many patients suffer falls because they do not receive the appropriate preventive care [40].

Almost all studies on the incidence of falls that we reviewed were in agreement with the benefits of applying preventive measures based on the risk identified and/or illness [13,15,16,18,20,22,30,32,39]. A systematic review by Avanecean et al. [35] indicated patient-centered interventions, in addition to tailored patient education, may have the potential to be effective in reducing fall rates in acute care hospitals.

With regard to the training process implemented, we observed that it was effective in achieving a reduction in falls; this shows that the process of assessment and risk detection is not always optimal (affecting the quality of care and patient safety), and that continuous and advanced training of nurses is essential. This is consistent with similar studies in different settings [8,9,23,24,41,42]; AbuAlRub and Abu Alhijaa [41] noted in their study with senior nurses that an advanced training intervention improved outcomes and reduced adverse events, including falls. In addition, we found that advanced training helps to detect patients at risk of falling, which allows specific strategies to be designed within the care plan to reduce or control risk. Some studies have also concluded that preventive education for cancer patients at risk of falling can reduce falls significantly [42].

This last reflection indicates that it is necessary to improve the clinical practice of nurses through advanced training. To do this, we will plan new practice models that influence the elements that increase the risk of falls, with evidence-based practices such as advanced and specific training in risk assessment [43]. Systematic reviews affirm that it is necessary to increase the concern of professionals, because this can reduce the risk of falls [44].

The implications of our study for professional practice include a reduction in the number of patient falls as a result of protocolizing an advanced assessment that includes specific evaluation of the risk of falls in hospitalized patients (such as having some type of catheter), as well as optimizing of the plan of care to be more adapted to these detected risks. Following the results of this investigation, a Systematic Assessment Procedure has been implemented in all areas of the hospital, indicating that it is seen as an excellent tool to reduce this adverse event and improve the quality of care.

It is, however, necessary to reflect on why not all nurses voluntarily adhere to this type of training program, because based on the available evidence and the results of our study, it is effective in reducing falls. The training level of nurses is an element that has generated ample evidence as a factor which can allow for the improvement of patient outcomes [45]. We believe that all nurses in hospital units with vulnerable patients should undergo such training, and that new and more extensive studies should continue to be carried out that will allow them to broaden their knowledge of the problem. Similarly, we consider it essential to explore new advanced training interventions focused on risks to patient safety; this will allow for an increase in evidence supporting improved training interventions and professional development.

## 5. Limitations

We believe that in this research the “Hawthorne effect” or “observer effect bias” could have occurred. The nurses, both from the intervention units and those belonging to the control group, could have changed their behavior in some way as a result of knowing that patients for whom they were responsible were being monitored.

On the other hand, not all nurses in the intervention group received training in systematic evaluation (84.6% of them were trained, and 66% of patients were evaluated), so there were patients from those units who did not undergo a systematic assessment of the risk of falling.

The study focused on improving the assessment process of nurses, thus improving the detection of patients at risk. Therefore, no exhaustive information was obtained about the pathological processes of the patients; in particular, not enough information was obtained about the patients’ baseline characteristics, nor the effect of the different independent variables on the results, such as surgical intervention.

Finally, the design of the study itself was a limitation, as we randomized the hospital units (since the training intervention was aimed at the nurses of the unit), and not the patients, as subjects of the study.

## 6. Conclusions

We found that the advanced training of nurses in fall prevention improves patient outcomes. In our study, the patients to whom the intervention was applied were less likely to fall, regardless of age and length of stay. The systematic assessment of the risk of a patient falling during the hospital processes has proved to be an effective intervention to reduce the incidence of falls, especially in the elderly, who have the most falls. It is, therefore, necessary to implement specific advanced training for all nurses and not as a voluntary training program. There is a need to further improve the evidence on clinical practices to ensure patient safety (such as fall risk prevention), especially with experimental studies.

## Figures and Tables

**Table 1:** Sample description by Study Group.

Variables	Totals *n* = 581 *n* (%)	Control Group *n* = 278 *n* (%)	Intervention Group *n* = 303 *n* (%)
Gender:			
Men	294 (50.6)	135 (48.6)	159 (52.5)
Women	287 (49.4)	143 (51.4)	144 (47.5)
Mean Age ± Standard Deviation	68.3 ± 16.2	66.78 ± 17.08	69.67 ± 15.32
Age Group:			
15–50 years	85 (14.6)	51 (18.3)	34 (11.2)
51–64 years	105 (18.1)	47 (16.9)	58 (19.1)
65–79 years	224 (38.6)	107 (38.5)	117 (38.6)
≥80 years	167 (28.7)	73 (26.3)	73 (26.3)
Nurse assessment on admission:			
Traditional Method	372 (69)	269 (96.8)	103 (34)
Systematic method	209 (31)	9 (3.2)	200 (66)
Risk assessment of falls on admission:			
Yes	213 (36.7)	10 (3.6)	203 (67.0)
No	368 (63.3)	268 (96.4)	100 (33.0)
Average Hospital Stay			
± Standard Deviation (days)	12.2 ± 9	13.71 ± 10.19	10.89 ± 7.49
Days interval:			
0 to 7 days	205 (35.3)	76 (27.3)	128 (42.4)
≥8 days	376 (63.7)	202 (72.7)	174 (57.6)
Mobility			
None (bed-to-armchair)	146 (25.1)	65 (23.4)	81 (26.7)
Unaided in room/bathroom	124 (21.3)	45 (16.5)	78 (25.7)
Unaided outside the room	311 (53.5)	167 (60.1)	144 (47.5)
Surgical intervention:			
Yes	270 (46.5)	42 (15.1)	228 (75.2)
No	311 (53.5)	236 (84.9)	75 (24.8)
Altered consciousness:			
Yes	59 (10.2)	41 (14.7)	18 (5.9)
No	522 (89.8)	237 (85.3)	285 (94.1)
Nutritional status:			
Risk and/or malnutrition	272 (46.8)	150 (54)	122 (40.3)
Normal nutritional status	309 (53.2)	128 (46)	181 (59.7)
Supply of Oxygen:			
Yes	119 (20.5)	50 (18)	69 (22.8)
No	462 (79.5)	228 (82)	233 (77.2)
Catheters (intravenous line, gastric, bladder tube, drainage):			
None	7	5 (1.8)	3 (1)
Has one catheter	263	205 (73.7)	96 (31.7)
Has 2 or more catheters	308	68 (24.5)	204 (67.3)

**Table 2:** Incidence of falls.

Variables	Falls	
	No *n* (%)	Yes *n* (%)
Gender		
Men	288 (50.2)	6 (85.7)
Women	286 (49.8)	1 (14.3)
Age		
15–50 years	85 (14.8)	0 (0)
51–64 years	104 (18.1)	1 (14.2)
65–79 years	221 (38.5)	3 (42.9)
≥80 years	85 (14.8)	3 (42.9)
Nursing Units		
General Internal Medicine	135 (23.5)	4 (57.1)
Neurology/Neurosurgery	137 (23.9)	2 (28.2)
Traumatology/Urology	206 (35.9)	0 (0)
Vascular Surgery/Nephrology	96 (16.7)	1 (14.2)
Groups		
Intervention	302 (52.6)	1 (14.3)
Control	272 (47.4)	6 (85.7)
Nurse assessment on admission		
Traditional Method	208 (36.2)	1 (14.3)
Systematic method	366 (63.1)	6 (85.7)
Risk assessment of falls on admission		
No	212 (36.2)	6 (85.7)
Yes	362 (63.8)	1 (14.3)
Hospital Stay (days)		
0–7 days	204 (35.5)	1 (14.3)
≥8 days	370 (64.6)	6 (85.7)
Mobility		
None (bed-to-armchair)	146 (25.4)	0 (0)
Unaided in room/bathroom	120 (20.9)	4 (57.1)
Unaided outside the room	308 (53.7)	3 (42.9)
Surgical intervention		
Yes	264 (46)	1 (14.2)
No	310 (54)	6 (85.7)
Altered consciousness		
Yes	59 (10.3)	0 (0)
No	515 (89.7)	7 (100)
Nutritional status on admission		
Risk and/or malnutrition	267 (46.5)	5 (71.4)
Normal nutritional status	307 (53.5)	2 (28.6)
Supply of Oxygen		
Yes	117 (20.4)	2 (28.6)
No	457 (79.6)	5 (71.4)
Catheter (intravenous, gastric/bladder, drainage)		
None	8 (1.4)	0 (0)
Has one catheter	294 (51.2)	7 (100)
Has 2 or more catheters	272 (47.4)	0 (28.6)

**Table 3:** Results of the logistic regression model.

	Estimate	Std. Error	OR *	Lower 95%	Upper 95%
Intercept	−6842	2581	0.001	0	0.088
Intervention Group	−2062	1054	0.127	0.013	0.821
Stay	−0.04	0.053	0.961	0.849	1044
Age	0.045	0.032	1,046	0.991	1119
WAIC	76,754	23,922			

* OR: Odds Ratio.
